# In this issue we will celebrate 50 years of the International Brazilian Journal of Urology

**DOI:** 10.1590/S1677-5538.IBJU.2024.02.01

**Published:** 2024-03-18

**Affiliations:** 1 Universidade do Estado do Rio de Janeiro Unidade de Pesquisa Urogenital Rio de Janeiro RJ Brasil Unidade de Pesquisa Urogenital - Universidade do Estado do Rio de Janeiro - Uerj, Rio de Janeiro, RJ, Brasil; 2 Hospital Federal da Lagoa Rio de Janeiro RJ Brasil Serviço de Urologia, Hospital Federal da Lagoa, Rio de Janeiro, RJ, Brasil

**Figure 1:**
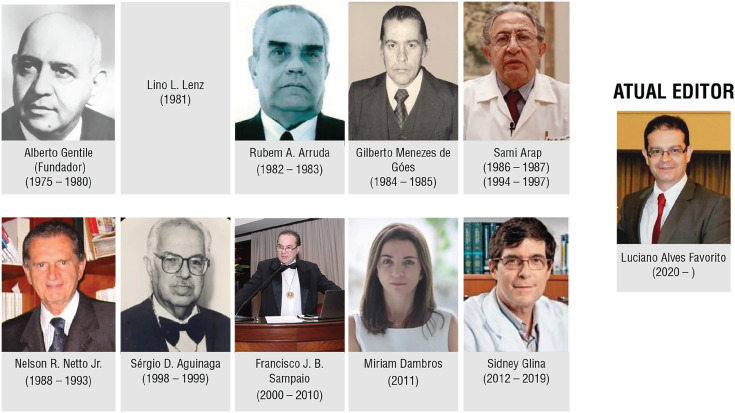
In this figure we can observe the operating time of the former editors of International Brazilian Journal of Urology in the last 50 years.

**Figure 2:**
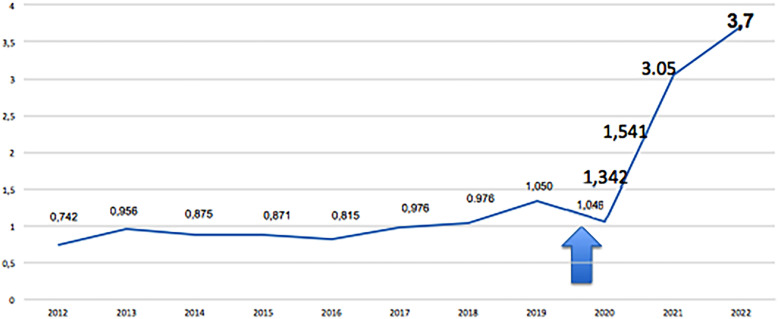
The figure shows the evolution of the impact factor of Int Braz J Urol in the last years.

The March-April number of *Int Braz J Urol* is the 27^nd^ under my supervision and is very special. In 2024 the Int Braz J Urol celebrate **50 years** of foundation. I would like to thank our former editors, doctors Alberto Gentile, Lino L. Lens, Rubem Arruda, Gilberto M. Goes, Sami Arap, Nelson R. Neto Jr., Sergio Aguinaga, Francisco Sampaio, Miriam Dambros and Sideny Glina. In Figure-1 we can observe the operating time of the former editors in the last 50 years. I highlight the importance of Prof. Francisco Sampaio that internationalized our Journal and Prof. Sidney Glina that adopted the submission system. In the cover of this edition, we can observe the new symbol of our Journal. In Figure-2 we observe the evolution of the impact factor of Int Braz J Urol in the last years. In 2023 we had the **impact of 3.7** the biggest of our history.

In this number the Int Braz J Urol presents original contributions with a lot of interesting papers in different fields: Robotic Surgery, Bladder Cancer, Uroanatomy, Hormonal reposition, Infection, Enuresis, ChatGPT in urology, Research in Urology, Endourology and Testicular Cancer. The papers came from many different countries such as Brazil, USA, Canada, Italy and Germany, and as usual the editor’s comment highlights some of them. The editor in chief would like to highlight the following works:

Dr. Glina and collegues from Brazil, presented in page 119 (1) a nice systematic review about statins and decrease of testosterone in men and concluded that the statins use causes a decrease in total testosterone, not enough to cause a drop below the normal range and also determines increase in FSH levels. No differences were found in LH, Estradiol, SHBG and Free Testosterone analysis.

Dr. Dutra and collegues from Brazil, presented in page 136 (2) a important systematic review about the treatment of monosymptomatic enuresis (MNE) in children and adolescents and concluded that Parasacral transcutaneous electrical nerve stimulation can reduce the occurrence of wet nights in children and adolescents with MNE. However, it is not a complete cure for the condition, except for one study that reported a 27% cure rate among patients. To determine the most effective protocol for this treatment, more high-qual- ity research is needed. Comprehensive evaluation of its effectiveness will require larger samples and more sessions.

Dr. Talizin and collegues from Brazil, performed in page 152 (3) a nice systematic review about the postoperative antibiotic prophylaxis for percutaneous nephrolithotomy and risk of infection and concluded that there is no benefit associated with the use of post- operative antibiotic prophylaxis until nephrostomy tube withdrawal in patients undergoing percutaneous nephrolithotomy (PCNL). We recommend that antibiotic prophylaxis should be administered only until the induction of anesthesia in PCNL.

Dr. Miranda e Morais and collegues from the Urogenital Resarch Unit from Brazil performed in page 164 (4) a nice narrative review about the kidney collecting system anatomy applied to endourology and concluded that the knowledge of intra-renal collecting system divisions and variations as the angle between the renal pelvis and lower infundibula, position of the calices in relationship with renal edge and the diameter and position of the calyces are important for the planning of minimally invasive renal surgeries.

Dr. Fu and collegues from USA performed in page 178 (5) an interesting study about the risk factors for perioperative outcomes in Intracorporeal Urinary Diversion (ICUD) and Extracorporeal Urinary Diversion (ECUD) with Robotic cystectomy and concluded that Robotic cystectomy with ICUD results in shorter hospitalizations and lower intraoperative transfusion rates compared to ECUD, without differences in operative time, high-grade postoperative complications, or readmission rates. These findings can inform clinical decision-making, highlighting ICUD as a potentially more favorable option in ap- propriate settings.

Dr. Braga and collegues from Brazil and Canada performed in page 192 (6) a nice study about the use of ChatGPT in Urology and its Relevance in Clinical Practice and concluded that ChatGPT simulated general knowledge on the researched topics. Regarding Enuresis, the provided definition was partially correct, as the generic response allowed for misinterpretation. For VUR, the response was considered appropriate. For pMU it was partially correct, lacking essential aspects of its definition such as the diameter of the dilatation of the ureter. Unnecessary exams were suggested, for Enuresis and pMU. Regarding the treatment of the conditions mentioned, it specified treatments for Enuresis that are ineffective, such as bladder training. Therefore, ChatGPT responses present a combination of accurate information, but also incomplete, ambiguous and, occasionally, misleading details.

Dr Beatrice and collegues from USA, Italy, Germany and Brazil performed in page 199 (7) a nice study about smoking Characteristics and Years Since Quitting Smoking of US Adults Diagnosed with Lung (LC) and Bladder (BC) Cancer: A National Health and Nutrition Examination Survey Analysis and concluded that BC patients exhibit a prolonged latency period between smoking cessation and cancer diagnosis compared to LC patients. Despite smoking status evaluation in microhematuria, current risk stratification models for urothelial cancer do not incorporate it. Our findings emphasize the significance of long-term post-smoking cessation surveillance and advocate for integrating smoking history into future risk stratification guidelines.

The Editor-in-chief expects everyone to enjoy reading this special number.
